# Clinical Importance of the Posterior Inferior Cerebellar Artery: A Review of the Literature

**DOI:** 10.7150/ijms.49137

**Published:** 2020-10-18

**Authors:** Hui-Lei Miao, Deng-Yan Zhang, Tao Wang, Xiao-Tian Jiao, Li-Qun Jiao

**Affiliations:** 1 Department of Neurosurgery, Xuanwu Hospital, Capital Medical University, No. 45, Changchun Street, Beijing 100032, China.; 2 School of General Practice and Continuing Education, Capital Medical University, Beijing 100069,China; 3 Peking Union Medical College and Chinese Academy of Medical Sciences, Beijing 100730, China.

**Keywords:** posterior inferior cerebellar artery, clinical importance, anatomy, aneurysms

## Abstract

The posterior inferior cerebellar artery (PICA), with its unique anatomical complexity, is of great clinical importance and involved in many diseases including aneurysm, ischemic stroke, neurovascular compression syndrome (NVCS), arteriovenous malformation (AVM), and brain tumor. However, a comprehensive systematic review of the importance of the PICA is currently lacking. In this study, we perform a literature review of PICA by searching all the associated papers in the PUBMED database hoping to provide a better understanding of the artery. The PICA has tortuous and variable course and territory, divided into 5 segments. Various aneurysms involving PICA were not uncommon, of which the treatment is challenging. The PICA infarct typically manifests lateral medullary syndrome (LMS) and is more likely to cause mass effects. The PICA frequently compresses the medulla and the cranial nerves resulting in various neurovascular compression syndromes (NVCS). Arteriovenous malformation (AVM) fed by PICA are associated with aneurysm and dissection which have high risk of rupture and worse outcome. PICA injured by head trauma can cause fatal SAH. VA terminating in PICA probably cause Bow hunter's syndrome (BHS). The PICA supplies many brain tumors and can be used in intracerebellar chemotherapy. The PICA can be exposed and injured during surgeries especially in telovelar approach, and it also plays an important role in bypass surgeries, hinting the surgical importance of PICA. In conclusion, PICA is very important in clinical practice.

## Introduction

**Figure 1:**
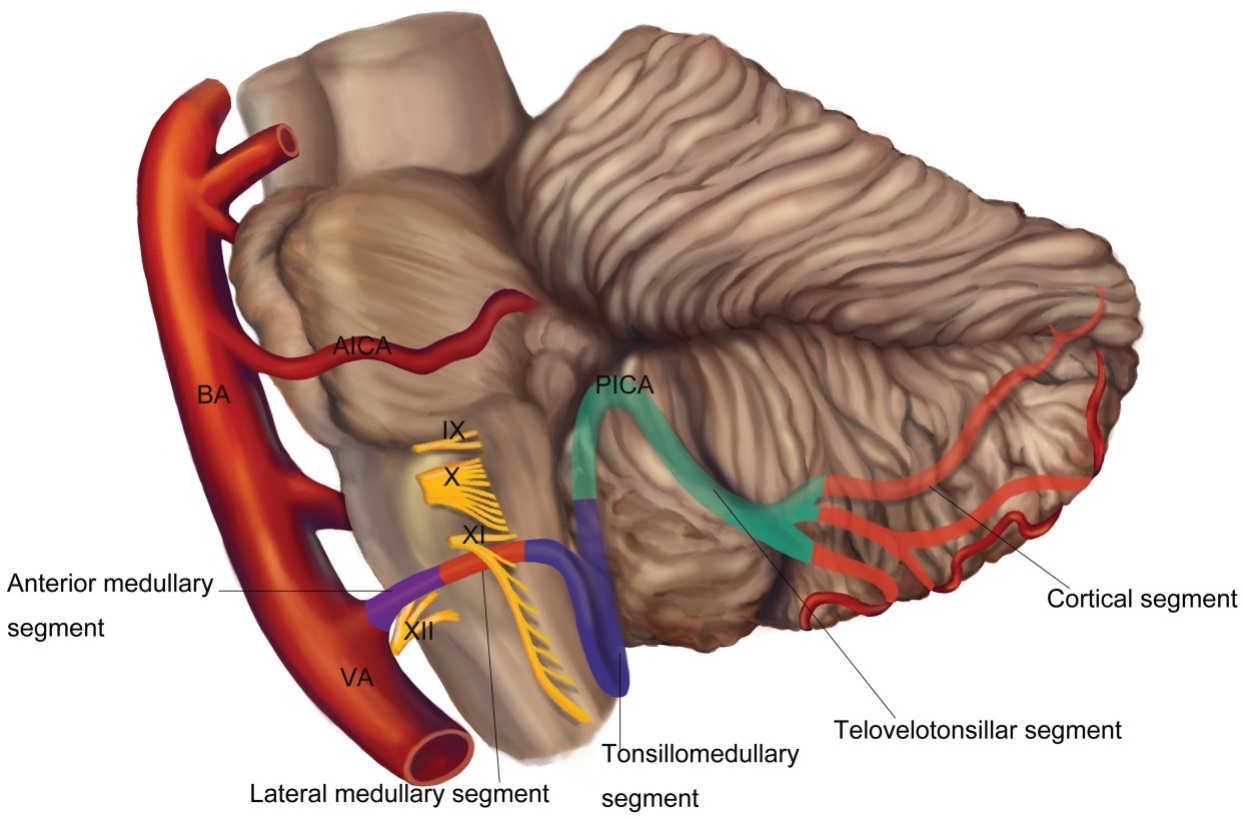
Normal segmental anatomy of the PICA.

The posterior inferior cerebellar artery (PICA) usually originates from the vertebral artery (VA) at an average distance of approximately 16 or 17 mm below the vertebrobasilar junction 1, 2. The VA, arising from the subclavian artery, is classically divided into 4 segments. The first three segments were extracranial and the fourth segment is entirely intracranial and terminates with forming the basilar artery (BA). It is the fourth segment of VA that gives off the PICA, which is the largest branch of VA 3-5. The trunk of PICA is divided into five segments (Figure 1): (1) the anterior medullary segment, which begins at the origin of the PICA and ends at the level of a rostrocaudal line that passing through the most prominent part of the inferior olive; (2) the lateral medullary segment, which extends from the level of the most prominent point of the olive to the level of the origin of the glossopharyngeal (CN Ⅸ), vagus (CN Ⅹ), and accessory (CN Ⅺ) rootlets; (3) the tonsillomedullary segment, which begins where the PICA passes posterior to the CN IX-XI and ends at the midpoint of the PICA's ascent toward the roof of the fourth ventricle along the medial surface of the tonsil; (4) the telovelotonsillar segment, which begins where the PICA ascends to the mid-level of the medial surface of the tonsil and ends where the artery exits the fissures between the tonsil, vermis, and hemisphere to reach the suboccipital surface; (5) the cortical segment, this segment begins where the PICA leaves the groove between the vermis, tonsil and hemisphere, and includes the terminal cortical branches 1, 6. The trunk of PICA gives rise to perforating, choroidal, and cortical branches 1. The PICA supplies the medulla, the choroid plexus and tela choroidea of the fourth ventricle, the tonsils, the inferior vermis, and the inferior aspects of the cerebellar hemispheres 1, 7. Furthermore, PICA mostly supplied the choroid plexus on the roof and the median opening of the fourth ventricle and gave the majority of branches of choroid plexus 8.

**Figure 2:**
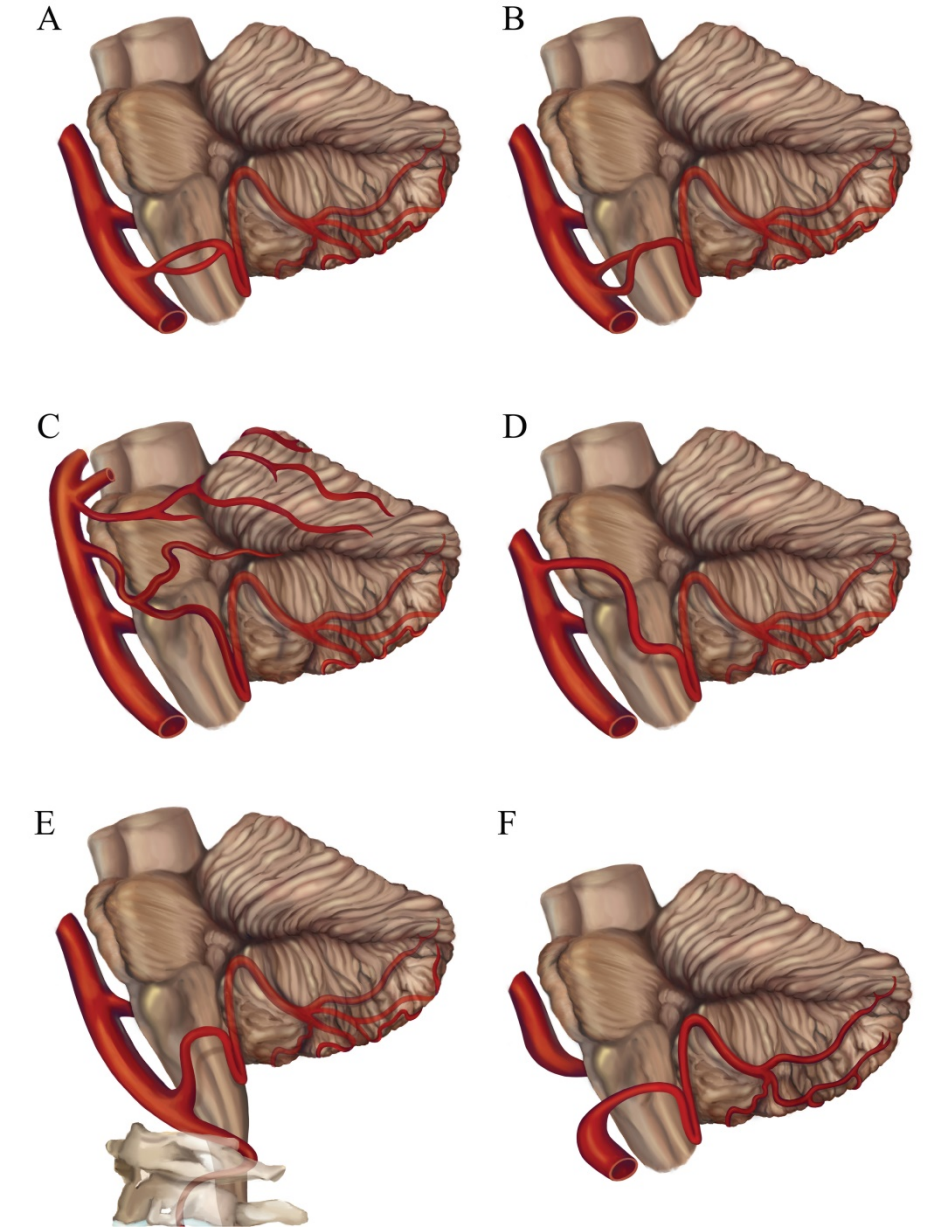
** The anatomic variants of the PICA.** (A) Fenestration of the PICA; (B) Double origin of the PICA; (C) The AICA-PICA variant; (D) The PICA originates from the BA; (E) The extradural origin PICA; (F) The VA terminates in the PICA. VA, vertebral artery; BA, basilar artery; AICA, anterior inferior cerebellar artery; IX, glossopharyngeal nerve; X, vagus nerve; XI, accessory nerve; XII, hypoglossal nerve.

The PICA shows a high frequency of variations. The PICA is observed to be hypoplastic in 15 - 32% of the cases 9, 10. Unilateral absence of PICA is reported in 6 - 26% of cases, absence bilaterally of PICA is reported in 2 - 3.6% of cases 1, 11-14. If one PICA is hypoplastic or absent, the ipsilateral anterior inferior cerebellar artery (AICA) or the contralateral PICA is larger and supplies the area normally nourished by the ipsilateral PICA 10. Duplication of the PICA occurs in 2 - 6% of hemispheres 1, 10, 15. Double origin of the PICA is a rare variation different from duplication, in those cases two distinct PICAs with separate origins converge distally. The reported prevalence of this variation is about 1.45% 16. This type of variation is closely related to aneurysm formation, and the incidence of aneurysms in double-origin PICA ranges from 50 - 71% 17. Fenestration is a rare but well-known arterial anatomic variation in which a segment of artery divides into two distinct lumens that reunite distally. The incidence of PICA fenestration is reported to be 0.3% 18. Some investigators hold the view that variations such as duplications and fenestrations were prone to the coexistence of vascular anomalies including aneurysms, vascular malformations, dissections due to the abnormal vascular structure and hemodynamics 5.The VA terminating in the PICA is reported in 2.8 - 7% patients, which is also one of the most common variations of VA 3, 18-20. This variation may have a detrimental impact on cerebral hemodynamics 21. The location of the origin of the PICA is extremely variable. The PICA origins at all points along the intradural VA are reported 1, 2. An extradural origin PICA from the VA is not rare. The frequency of an extradural origin of the PICA ranges from 0.4% to 20.8% 1, 4, 12, 18, 19, 22-25. The extradurally located PICAs are exposed to injury during posterior approaches to the lower brainstem and upper cervical spine. The PICA may also arise from the BA. Overall, a PICA arising from the BA appears in 6 - 11 % of cases 12, 20. But Icardo et al 9 reported that the PICA originated from the BA in 53.33% cases, of which 25.0% were directly from the lower third of the BA and 28.33% had a common trunk with the AICA. The AICA-PICA common trunk anomaly is one of the most common variants in the posterior circulation, and it has a prevalence of 20 - 24% based on retrospective studies 26. Recognizing this anomaly is important because inadvertent injury to this variant at its origin during surgery would result in strokes involving both PICA and AICA territories. PICAs origins from other arteries such as internal carotid artery, primitive hypoglossal artery, primitive trigeminal artery and posterior meningeal artery have also been reported 27-30. Rarely, the variation of primitive trigeminal artery (PTA), which is the most common persistent carotid-basilar anastomose, could originate from internal carotid and continue as PICA 31. The anatomic variants of PICA are shown in Figure 2A-F.

**Figure 3:**
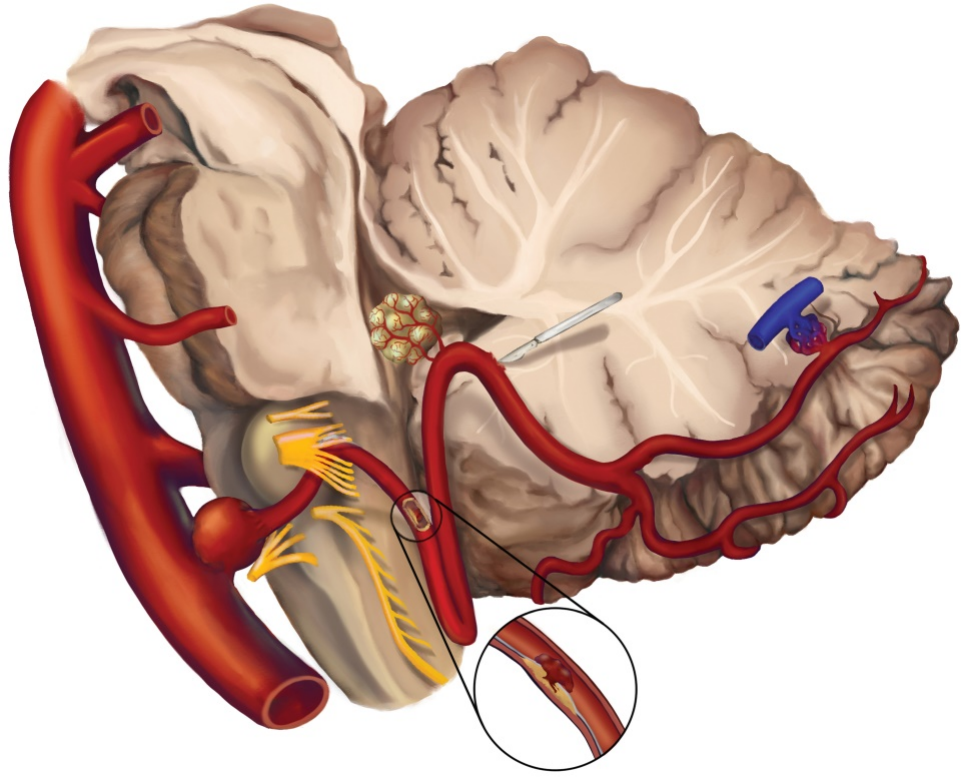
** The overview of the diseases involving PICA.** Common diseases including aneurysm, ischemic stroke, neurovascular compression syndrome (NVCS), brain tumors supplied by PICA, surgical injury, arteriovenous malformation (AVM) on PICA are illustrated.

Therefore, the PICA is of great clinical importance with its unique anatomical complexity and is involved in many diseases, including ischemic stroke, aneurysm, neurovascular compression syndrome (NVCS), arteriovenous malformation (AVM), brain tumor, etc. Besides, the PICA is exposed in many surgery approaches and vulnerable to damage. However, a comprehensive review of the importance of the PICA is currently lacking. A systematical search based on the anatomy and clinical significance of PICA was performed via PubMed database. There were no restrictions setting on publication time and the types of paper during the searches. Studies in language other than English were excluded. A total of 1128 publications has been searched after removing duplications. In this paper, we performed a comprehensive review of most of the representative literatures to increase the recognition of the role of the PICA. Figure 3 shows the overview of the diseases of PICA.

## Aneurysm

The most common disease involving PICA is aneurysm. About 1.4 - 4.5% of all intracranial aneurysms originate from PICA 32-34. PICA aneurysms are heterogeneous in both location and morphology, which could arise in any of the 5 segments of PICA while the VA-PICA junction is most frequently involved. Distal PICA aneurysms only account for less than 30% 35, 36. Morphologically, PICA aneurysms display saccular, dissecting, and fusiform pattern. Richard et al reported that most of the ruptured PICA aneurysms were saccular whereas the PICA was more inclined to form non-saccular aneurysms than other arteries 37. Although the dissecting aneurysms account for less than 1/3 of all the PICA aneurysms, the specificity in clinical features and treatment options and worse course call for special attentions 37-40. Unusual aneurysms located in AICA-PICA variant, the bifurcation of persistent primitive hypoglossal artery (PPHA)- PICA, the fenestration of the PICA, the double origin of the PICA and extracranial PICA were also reported 41-45. Giant aneurysms and multiple peripheral aneurysms were also found in previous studies 46-48.

PICA aneurysms have a broad spectrum of clinical and radiological manifestations. Most patients present symptoms associated with subarachnoid hemorrhage (SAH). The headache is more common in occipital area and neck, compared with supratentorial aneurysms 49-51. More than 83% of the patients with a ruptured aneurysm developed intraventricular hemorrhage (IVH) and obstructive hydrocephalus as PICA was adjacent to the fourth ventricle anatomically 52. Localizing symptoms appeared when cranial nerves and structures of brain stem and cerebellum were involved due to compression or infarcts 53-55. Proximal PICA dissecting aneurysms tended to cause infarcts compared with other PICA aneurysms 40, 56, 57. The clinical features of brain infarcts will be described in the following part. Although abducens nerve was not directly adjacent to PICA, ruptured PICA aneurysm could result in isolated abducens nerve palsy, which was possibly associated with the hemodynamics of aneurysmal rupture instead of direct compression 58. Burkhardt et al demonstrated ruptured PICA aneurysm was an independent risk factor of abducens nerve palsy 59. Giant aneurysms might be misdiagnosed as tumors of posterior fossa due to similar symptoms and imaging features which were sometimes indistinguishable even with magnetic resonance angiography (MRA) and digital subtraction angiography (DSA) 46, 47. Computed tomography angiography (CTA) is insensitive to the distal PICA aneurysms resulting from the thin lumen of PICA. Yuan's retrospective study included 18 confirmed cases with distal PICA aneurysm; only 2 of them were identified by CTA 60.

PICA aneurysms have a high tendency to rupture even small in size due to its relatively fragile vessel wall 61. The average size of ruptured PICA aneurysm was about 6 mm, with a minimum of 1 mm 34, 36, 37. The rupture rate of PICA aneurysms ranged from 77% to 88% in some large retrospective studies 33, 34, 36, 62. More than 20% of the ruptured intracranial aneurysms were from PICA aneurysms though with low incidence 32, 37. The mortality resulting from recurrent hemorrhage within 48 hours of ruptured PICA aneurysms was 3 times higher than anterior circulation aneurysms 63, 64. As a consequence, the early identification and treatment are of vital importance. For unruptured intracranial aneurysms, patients with documented enlargement during follow up, a history of SAH or a family history of intracranial aneurysms are recommended treatment. However, there is still no consensus regarding the treatment indications for PICA aneurysm due to its rarity and diversity of location and morphology.

Endovascular treatment (EVT) has been the dominant treatment strategy of PICA aneurysms recently, which was applied in 55.4% of the ruptured PICA aneurysms 32. EVT reduces the risk of direct brainstem injury and anesthesia related complications, which is more preferable for patients with poor clinical status 34. EVT strategies such as stand-alone coiling, stent-assisted coiling, microcatheter-assisted coiling, flow diversion, parent artery occlusion with coils were successfully used to treat PICA aneurysms 33, 65-73. The treatments of PICA dissecting aneurysms were prone to require vessels sacrifice 38. Nevertheless, EVT related complications including intraprocedural ruptures and new-onset infarcts are not rare 33. Chen's study even reported that the morbidity of EVT related imaging infarcts was up to 33%, most of them didn't show apparent symptoms fortunately 65. A meta-analysis suggested PICA aneurysms treated with EVT had a higher recurrence rate and lower occlusion rate than those treated with surgery 35. Robert et al also reported 29.4% of the patients who underwent EVT required retreatment due to aneurysm recurrence 72. The safety and efficacy of EVT still need to be improved.

Broadness of the neck, non-saccular morphology, small caliber of the PICA, small angulation between the PICA and VA, the presence of vasospasm in ruptured cases, and tortuosity of the PICA sometimes make endovascular approaches very difficult. In cases of massive IVH or hydrocephalus, EVT would never be able to offer decompressive effect. Hence, in the actual endovascular era, surgical treatment remains a valid option for 28% of PICA aneurysms while 4.8% of non-PICA aneurysms 32. Microsurgical treatments including clipping, trapping, resection, and a variety of bypasses procedures have been used to treat PICA aneurysms and acceptable clinical outcomes have been obtained 61, 74-80. However, the deep location of the PICA aneurysms, the intimate relationship of PICA aneurysms with the lower cranial nerves, as well as the presence of subarachnoid hemorrhage around the brainstem make microsurgical dissection of these aneurysms difficult. Previous reports noted that new cranial nerve palsies occurred at an incidence of 20 - 60% after surgical treatment 81. For many years, controversy has raged over the pros and cons of the various surgical routes to approach PICA aneurysms. The far-lateral approach is a standard approach for PICA aneurysms clipping 75, 77, 78, 82, 83.

Compared to saccular aneurysms, dissecting aneurysms in PICA had worse clinical outcomes including longer hospital stays, more likely to receive extraventricular drain and ventriculoperitoneal shunt, as well as more ischemic infarcts when treated with EVT. Therefore, bypass surgery was recommended to patients with dissecting PICA aneurysms 38, 84, 85.

## Ischemic stroke

PICA plays an important role in the blood supply of cerebellum, and the PICA territory is the most common area of cerebellar ischemic stroke of, accounting for 40% 86. The cerebellar branches and choroidal branches of PICA have abundant anastomoses with superior cerebellar artery (SCA) and AICA while medullary branches are lack of anastomoses. Thus, the most frequently involved ischemic region of the PICA infarct is the dorsal lateral medulla supplied by the medullary branches. This causes lateral medullary syndrome (LMS), which will be detailly described later. Except LMS, the infarcts of the medial branch of the PICA (mPICA) could lead to vertigo, nausea, vomiting and postural instability because of inadequate blood flow of vestibulocerebellum. Isolated vertigo could occur in isolation 87, 88. The infarcts of the lateral branch of the PICA (lPICA) could cause dysmetria and hypotonia of ipsilateral limb 89. Rarely, the PICA infarcts might present acute hearing loss when PICA or basal artery gave rise to the internal auditory artery 90, 91.

Various pathogenesis of PICA infarcts includes large artery atherosclerosis, cardiogenic embolism, in situ branch artery disease of PICA and VA dissection 92. Lee et al reported that greater angle between PICA and VA was more likely to result in cardioembolic occlusion of PICA 93. Although patients with PICA dissection tended to hemorrhage compared with ischemia 94, Junpei et al still found that isolated PICA dissection caused 6% of the PICA region infarcts by reviewing 167 cases, equal to VA dissection, which hinted the undervalued clinical importance of PICA dissection 95. Jeong et al reported a rare case of PICA infarct after cervical chiropractic manipulation 96. Extradural origin of PICA could be injured accidently by the cervical surgery 97. As a result, the history of mechanical injury and surgery should be noticed when faced with PICA infarct of unknown origin.

As known computer tomography (CT) can rarely identifies early-stage infarcts and the sensitivity of diagnosing infarcts in posterior fossa is even lower due to the artifacts of skull base 98. Magnetic resonance imaging (MRI) is preferable in the early diagnosis of infarcts and T2-weighted images (T2WI) are more sensitive than fluid-attenuated inversion recovery (FLAIR) in the identification of posterior fossa infarcts 99, 100. Diffusion-weighted magnetic resonance imaging (DWI) is widely used in early infarcts identification. However, vertebrobasilar strokes were prone to show false-negative pattern in DWI probably because of the tiny lesions and magnetic susceptibility artifacts 101. What's more, a three-component bedside oculomotor examination called HINTS is superior to MRI in the detection of mPICA infarcts. HINTS included horizontal head impulse test, nystagmus and test of skew, which indicated the importance of specific physical examinations 102, 103.

Treatment strategies vary from the etiology of infarcts. Similar to other ischemia strokes, intravenous recombinant tissue-type plasminogen activator (rt-PA) is recommended in thromboembolic PICA infarcts 104. Selective intra-arterial rt-PA has been used in acute PICA infarcts recently 105. For those infarcts caused by dissection, the efficiency and risk of hemorrhage of rt-PA were still uncertain 94. All patients require secondary stroke prevention in chronic stage 95, 106. Catastrophic complications such as increased intracranial pressure and obstructive hydrocephalus should be monitored. As PICA locates in the cramped posterior fossa, the infarct tissues of PICA region and surrounding edema take up larger space within posterior fossa, leading to medulla compression and hydrocephalus. According to Koh's study involving 90 patients with isolated cerebellum infract, 60% of the patients who suffered mass effect had PICA territory infarcts 107. Drainage of cerebrospinal fluid and decompressive craniectomy could be applied if need. However, the outcome of space-occupying cerebellar infarction after different treatment strategies was still unclear 108-110.

### Lateral medullary syndrome

LMS, also named Wallenberg syndrome or posterior inferior cerebellar artery syndrome, is due to damage to the lateral part of medullary oblongata caused by vascular events. The most frequently involved vessels are the PICA or the VA 111, 112. LMS is the most prevalent posterior circulation ischemic stroke syndrome and is the typical presentation of PICA territory infarcts. More than 20% of ischemic strokes appeared in posterior circulation and half of them manifested LMS as estimated 112, 113. Anatomically PICA is the relevant supplying artery of LMS. However, previous studies demonstrated that the VA which gave off PICA was the most responsible artery, accounting for 67%, followed by PICA (10%). Although atherosclerosis was still the most common cause of vascular events leading to LMS, PICA occlusion causing LMS was thought to be more relevant to cardiogenic embolism compared with other vessels 111, 114. Rarely, a fraction of isolated PICA dissection cases could present LMS 111. PICA dissecting aneurysms resulting in LMS was also reported 57. Razak et al. also considered extradural origin of PICA as a risk factor of LMS 116.

The clinical manifestations of LMS are various depending on the exact location of the damage. Vertigo with nystagmus, nausea and vomiting appear due to vestibular nucleus and vestibular-cerebellar connections involvement. Dysphagia, dysphonia and dysarthria, sometimes combined with ipsilateral loss of gag reflex and hiccup ascribe to defect of nucleus ambiguus, glossopharyngeal and vagus nerve, which is more significant in LMS patients associated with the evaluation and follow-up care. Ipsilateral ataxia is correlated with cerebellar peduncles, spinocerebellar fibers and inferior cerebellar hemisphere. Ipsilateral Horner syndrome shows decreased pupil size, a drooping eyelid and decreased sweating on the ipsilateral face due to sympathetic fibers defects. Impairment of pain and thermal sensation on the ipsilateral face and on the contralateral trunk and limbs are associated with impaired nucleus spinalis nervi trigemini or its spinal tracts, and spinothalamic tracts respectively. Different combination of these symptoms above can be found in LMS patients while more than 90% of the patients have sensory symptoms, which are the most frequent manifestations. These symptoms can appear either acutely or gradually, progressing over several hours to several days. The ratio of sudden and chronic course is about 3 111, 114, 117.

The diagnosis and treatment is similar to any ischemic stroke. In particular, dysphagia management and speech therapy assessment should be included for patients with corresponding symptoms. Overall LMS patients have a better prognosis than most other stroke syndromes, even those with severe dysphagia 114, 117.

## Neurovascular Compression Syndrome

NVCS covers a wide range of diseases caused by vessels compressing a variety of neural structures, and it is clinically characterized by functional disturbances of these effected structures 118, 119. The PICA frequently forms complex loops on the side of the brainstem, which distorts the medulla and the cranial nerves and results in various NVCSs.

### Glossopharyngeal Neuralgia (GPN)

GPN is characterized by a sudden onset of lancinating pain in the posterior pharynx, base of tongue, tonsillar region, or deep ear, that is, the sensory distribution of the auricular and pharyngeal branches of the CN IX, with an incidence of 0.7/100 000 per year 120, 121. The PICA is the primary culprit artery of GPN due to its close relationship with CN IX. Xia et al reported in nearly 90% of the patients, the CN IX was compressed by PICA, solely (72.3%) or combined with other vessels (17.2%) in their cohort involving 228 GPN patients 116. Furthermore, the neurovascular conflict involving PICA and both CN IX and CN Ⅹ was also reported, which was referred to as vago-glossopharyngeal neuralgia 122. Patients with vago-glossopharyngeal neuralgia manifested with not only GPN syndrome but also symptoms associated with excessive parasympathetic vagal outflow such as bradycardia and asystole 123, 124.

### Hemi-Laryngopharyngeal Spasm (HeLPS)

HeLPS is a newly defined rare syndrome associated with the compression of the CN X. Patients with HeLPS presented episodic throat contractions and grievous feeling of chocking that increased in severity, frequency, and duration over years. The syndrome was easily misdiagnosed as psychiatric illness or laryngeal diseases. Cases with HeLPS have been reported in at least 4 languages since 1926, which hinted the possibly underestimated incidence 125-127.

### Hemifacial Spasm (HFS)

HFS is the most common neurovascular syndrome characterized by paroxysmal, involuntary twitching of facial muscles in the region innervated by ipsilateral facial nerve (CN Ⅶ), with a prevalence of 7.4/100000 in men and 14.5/100000 in women 128, 129. Although AICA has the closest relation with CN VII anatomically, actually the tortuous PICA is the most frequent offending vessel in HFS, accounting for 47.2%, followed by AICA 130, 131. Chung et al noted that PICA compression was prone to left-sided symptoms 132.

### Trigeminal neuralgia (TN)

TN is an paroxysmal, lancinating and annoying facial pain syndrome occurring in the region innervated by the trigeminal nerve (CN V). The most common offending vessels are the SCA, AICA and vertebrobasilar trunk 133,134. Very rarely, TN could result from the PICA associated with the PTA as mentioned before 31. Several cases demonstrated that the artery that originated from the PTA and terminated as the sole PICA caused TN 134,135.

### Medullary Compression Syndrome (MCS)

MCS is the syndrome associated with the compression of medulla, mainly manifesting as hypertension. The PICA was reported as the dominant offending vessel 136-138. As known, the ventrolateral medulla plays an important role in the regulation of tonic cardiovascular reflexes 139-141. Therefore, the compression of the ventrolateral medulla by the PICA was considered as an important cause of essential hypertension 136, 138. Besides hypertension, dysesthesia, dysarthria, and hemiplegia could occur in MCS patients 142. Simpson et al reported a rare case that presented hemibody pain, MRI demonstrated indentation and displacement of the lower part of contralateral medulla by a loop of PICA 143.

Other NVCSs such as geniculate neuralgia, trigeminal neuralgia and vestibular paroxysm could result from compression of the corresponding cranial nerve by PICA in a few cases 144-146. Multiple cranial neuropathy caused by the offending PICA were also described, in these cases, patients presented with simultaneous trigeminal neuralgia and HFS or concurrent HFS and GPN or trigeminal neuralgia and GPN 147, 148.

The pathophysiology mechanisms of these NVCSs are similar, which includes demyelination induced by pressure and ectopic generation and ephaptic transmission of the impulses along the compressed nerve 121, 149. The diagnosis of these diseases mainly depends on specific clinical manifestations and MRI evidence. Microvascular decompression (MVD) is a well-established treatment for NVCS 150. High effective rates and good clinical outcomes have been reported with MVD in patients with these diseases above 120, 126, 148, 151.

## Arteriovenous malformation

AVM caused by congenital dysplasia of mesoderm vessels is a group of vasculatures that consist of feeding arteries, abnormal vascular nest and draining veins, with a detecting rate ranging from 1.10 to 1.4 per 10000 person-years 152. 5-9% of them are posterior fossa AVMs 1. PICA could serve as the feeding artery of posterior fossa AVMs 153-156. The most common clinical manifestation of posterior fossa AVMs was hemorrhage; some patients also presented seizure, headache, and dysphoria et al 153. Strikingly, feeding arteries aneurysms associated with AMVs drew attention due to the increasing risk of rupture and hemorrhage. It was supposed that the formation of aneurysms was probably associated with the high flow, which resulted from AVMs or the congenital impaired vessel walls 154, 157, 158. The PICA is more prone to form aneurysms associated with AVMs because its tortuous anatomic course more likely leads to hemodynamic abnormity 159. Kaptain et al presented 81% of the PICA aneurysms associated with AVMs located in the distal segments of PICA 154, David's study showed distal PICA aneurysms were frequently related to small AVMs 160. Furthermore, as proved, the risk of aneurysms rupture of PICA was significantly higher than AVMs rupture in PICA-AVM-aneurysm complex 157. Besides aneurysms, dissection of the PICA distal segment causing subarachnoid hemorrhage (SAH) associated with AVM was also reported. The possible pathogenesis was the high flow rates and shearing force in the highly tortuous segment 155.

Hemorrhagic stroke caused by posterior fossa AVMs and their associated diseases had worse outcome compared with supratentorial AVM, which hinted the importance of early diagnosis and treatment 153. DSA is the golden standard of diagnosis. Once diagnosed, the treatment strategies consist of microsurgical resection, endovascular embolization, stereotactic radiotherapy and the combination of these options. Observation is also a choice for those patients with mild symptoms 153. What's more, the treatment of PICA aneurysms and dissection associated with AVMs were still controversial. Some studies demonstrated AVM should be treated primarily because the associated diseases would cure after the removal of AVM 154, 155, 161. Other studies proposed the treatment of aneurysms should be prior due to the higher risk of rupture 154, 157, 162. In some cases, the PICA aneurysm and AVM could be treated simultaneously, with surgery or endovascular embolization 158, 162.

## Other diseases

### Traumatic PICA injury

Trauma to the head may cause various injuries including blood vessels injuries. PICA can be injured manifesting traumatic aneurysms, traumatic arteriovenous fistula, and direct rupture 163-170. Head trauma can result in acceleration and deceleration of the cerebellum, forming shearing forces leading to vessels torsion, overstretching and tearing, which is the possible mechanism of the injury of PICA 163, 166, 167, 169.

Traumatic aneurysms account for less than 1% of intracranial aneurysms and 10% of them involve posterior circulation, which is the most frequent PICA injury. The formation of traumatic aneurysms is related to blunt or penetrating injury and mostly associated with skull fractures. Children and males are prone to suffer from traumatic aneurysms because of higher trauma incidence 163, 167, 171. Traumatic aneurysms can be clarified as true aneurysms, false aneurysms, and dissecting aneurysms. The most common type is false aneurysm 172. The aneurysms mostly locate in the origin of PICA and proximal PICA 164-166. About 50% of the traumatic aneurysms rupture and cause catastrophic outcomes. The mortality rates range from 32% to 54%. Most of the ruptures are delayed onset and the average time from injury to rupture was 14-21 days, rarely several hours or several years. Delayed SAH is the most common clinical manifestation of PICA traumatic aneurysms rupture. DSA is still the golden standard for detection 163-166. However, the differential diagnosis of the rupture of traumatic aneurysms and pre-existing aneurysms is hard. Pre-existing aneurysms should be strictly excluded by the comparation of DSA results before and after trauma, which is extremely difficult. During clinical practice, the history of head trauma, young and male patients, the location of the aneurysms near falx edge and far away from branching points all support the diagnosis of traumatic aneurysms 163, 166. When the diagnosis is confirmed, the early intervention to PICA aneurysms is recommended.

Besides aneurysms, traumatic arteriovenous fistula of PICA was rarely reported after penetrating injury, which was cured by endovascular embolization 168. Cases with direct rupture of PICA causing death were also reported. The confirmation relied on autopsy after excluding aneurysms, atherosclerosis and medionecrosis 169, 170.

### Bow hunter's syndrome

Bow hunter's syndrome (BHS), officially named rotational VA occlusion, is a rare disease caused by mechanical and reversible occlusion or compression of the VA and lack of blood supply when rotate or stretch the neck 173-175. BHS often occurs in males in their sixties. The blood supply of the posterior circulation is commonly defective, the most common reason is VA hypoplasia and the VA terminating in PICA followed 173, 174. The clinical manifestations vary from mild vertigo to medulla stroke. Jost's study summarizing 126 cases showed syncope, drop attacks, vertigo, dizziness and impaired vision were more common symptoms, while hemiparesis, numbness, near loss of consciousness and headache and so on were less common 173. The final diagnosis depends on DSA which can reveal the stenotic or compressed arteries when rotate the neck. Recently, transcranial doppler (TCD) sonography has been used in the evaluation of the disease 174. Due to the limited number of patients, there is no agreement on standard treatment. Most of the patients underwent decompression and/or fusion surgery. A few patients chose conservative therapy including aspirin and cervical collar which could restrict the rotations of neck. Several cases were treated with endovascular intervention 173, 174. Rarely, one patient without obvious compression site received PICA-to-PICA in situ bypass surgery to rebuild the blood supply 176.

### Brain tumors

The PICA can supply many brain tumors including hemangioblastoma, ependymoma, subependymoma, papillomas of the choroid plexus and meningioma 8, 177-180. Patel et al reported a patient whose retromedullary hemangioblastoma was misdiagnosed as PICA aneurysm and angiography showed supply from PICA. Endovascular embolization failed and microsurgical specimen revealed the diagnosis of hemangioblastoma, which hinted the rare differential diagnosis of PICA aneurysms 177. Yan et al reported that a meningioma at craniocervical junction was adhering to VA and PICA. Meningioma in this area was mostly supplied by meningeal branches of VA, rarely by PICA. The preservation of VA and PICA was very important in the dissection surgery 178. The fourth ventricle tumors supplied by PICA and its branches deserved attention, which could be adjacent to and even encase the PICA. The approach to the fourth ventricle tumors and the microsurgical dissection should be extremely meticulous to avoid PICA injury 179, 180. The association between tumors and vessels should be evaluated by image examination such as DSA, CTA and MRA before operation if need. Besides surgery, PICA recently was used to treat lung adenocarcinoma with solitary cerebellar metastasis through microcatheter interventional therapy combined with bronchial artery, which showed safety and short-term efficacy. This new strategy benefited from the small space of posterior fossa and the direct saturation of the drug of cerebella tumor using PICA, which increased the local drug concentration 181. In conclusion, PICA is essential for the diagnosis and treatment of some brain tumors and should be protect carefully.

## Surgical exposure and application of PICA

Due to a great number of adjacent structures and complexity of the anatomy, even multiple variations existing, the PICA is quite possibly injured in surgeries. The spectrum of the consequence varies from spasms of branches to catastrophic occlusion and hemorrhage 182. The damaged locations probably make a difference depending on the number of anastomotic branches. The most mentioned surgical approach that may injure PICA is the telovelar approach through cerebellomedullary fissure corridors to the fourth ventricle, which can be utilized in fourth ventricular tumors resection, as well as arteriovenous malformation and aneurysms of PICA 183-185.

The telovelar approach is carried out by suboccipital craniotomy, cerebellar hemispheric and tonsillar retraction, cerebellomedullary fissure completely opening, and the uvulotonsillar and medullotonsillar space dissection sequentially, sometimes followed by the tela choroidea and inferior medullary velum opening depending on surgical require 186. The telovelotonsillar segment of PICA is mostly relevant to the approach thus it is mostly likely to be injured, followed by tonsillomedullary segment 187. Mechanical injury of them may cause PICA spasm, leading to ischemia and edema of dentate nucleus, which is associated with postoperative cerebellar mutism from several days to several months 188. Rarely, during the retraction of tonsil the branches of PICA supplying pneumotaxic center of medulla can be damaged, causing temporary or permanent respiratory arrest 1, 186. In addition, the occlusion of PICA due to the surgery of fourth ventricle may cause corresponding manifestation such as LMS 182. Therefore, it's of vital importance to protect the PICA carefully during operations, and utilizing endoscope can provide excellent visualization of PICA in order to reduce injury if necessary 186, 189. Besides telovelar approaches, the posterior fossa decompression treatment of Chiari I malformation, a congenital aplasia that cerebellar tonsils extends below the foramen magnum and into the upper spinal canal with or without symptom, makes the PICA under risk. Thus, delayed aneurysm probably forms during intradural exploration 190. Furthermore, Nassr et al reported a rare case who had PICA distribution stroke due to injury of a variant PICA. The PICA was oppressed by C1 lateral mass screw after an occiput-C6 posterior spinal instrumentation with bilateral C1 lateral mass screw placement. Radiographic examinations showed that the left PICA arose between the C1 and C2 transverse foramena as mentioned above and went exactly posterior to the C1 lateral mass screw 191. Won et al reported a similar case of extracranial origin of PICA 97. Lateral mass screw fixation of the atlas was widely used in upper cervical spine surgery; these reports recommended that surgeon concern about the PICA anatomic variations to reduce complications.

Bypass surgeries often include PICA for revascularization, which were mainly applied in the treatment of those intractable PICA aneurysms such as giant aneurysm, aneurysm involving VA or the origin of PICA 192, hinting the surgical importance of PICA. Rarely, PICA-PICA in situ bypass can also be used in the treatment of BHS 174. The bypass options can be divided into intracranial-intracranial (IC-IC) bypass and extracranial-intracranial (EC-IC) bypass 79, 193. The former incudes in situ bypass with PICA-PICA bypass, reimplantation of the PICA to the proximal fourth segment of the VA and PICA reanastomosis after aneurysm excision 193. The later mainly refers to occipital artery (OA)-PICA bypass. The caudal loop of PICA is recognized as the optimal receiving point of the bypass. However, as for those cases whose caudal loop are absent or in high position, accounting for about 28% 194, it is difficult to conduct the bypass directly thus alternative anastomosis sites should be explored through far-lateral approach 195. Kim et al demonstrated successful OA-high-riding PICA bypass in 2 cases with anatomical variations of caudal loop 196. Using radial artery interposition graft to realize the bypass of third segment of the VA and PICA might treat distal PICA aneurysms when other options were unsuitable 197.

In conclusion, PICA is easily injured during surgeries because of its special position, tortuous course and anatomical diversity. Surgeons need focus on the variations before the surgery and operate prudently, take advantage of endoscope when necessary to preserve PICA as possible. The anatomic features and course of PICA also play important roles in bypass surgeries, which emphasize the surgical importance of the vessel in the treatment of complex aneurysm and other rare diseases.

## Summary

As the largest branch of VA, the PICA supplies critical regions of the medulla, fourth ventricle and cerebellum. The PICA can be involved in many diseases, including ischemic stroke, aneurysm, NVCS, AVM, and brain tumor etc. During surgeries associated with the telovelar approach, the PICA medulla segments should be preserved to prevent the infarction of the critical territory supplied by PICA. PICA frequently forms complex loops on the side of the brainstem, which distort the medulla and the cranial nerves and result in various NVCSs, MVD is a highly effective first-line choice for treatment. In ischemic strokes, rapid treatment is necessary to avoid obstructive hydrocephalus. Posterior fossa AVMs associated diseases including aneurysm and dissection involving PICA should be focused because of their high risk of rupture, worse outcome and treatment options. PICA can be injured by head trauma, which causes fatal SAH and should be early diagnosed and treated. VA terminating in PICA probably causes BHS. PICA supplies many brain tumors and can be used in intracerebellar chemotherapy. Thus, the PICA is the vessel of vital importance in practice.

Table 1 listed the outline and key important points of the clinical importance of PICA.

## Figures and Tables

**Table 1:** Outline and key important points of the clinical importance of PICA

Outline	Key points
Anatomy	The PICA is the largest branch of VA that supplies critical regions of the medulla, fourth ventricle and cerebellum. It can be divided into 5 segments containing anterior medullary, lateral medullary, tonsillomedullary, telovelotonsillar, and cortical segment. It has close relationship to lower CN.
Aneurysm	PICA aneurysms are heterogeneous in both location and morphology. The dissecting PICA aneurysms need more attention. PICA aneurysms have a wider clinical and radiological manifestations than other aneurysms. It is challenging to treat PICA aneurysms by both microsurgical treatments and EVT.
Ischemic stroke	The PICA infarct typically manifests LMS and etiologically more relevant to cardiogenic embolism. Both intravenous and selective intra-arterial rt-PA may take effect in PICA occlusion. PICA infarction is more likely to cause mass effects compared with other vessels.
NVCS	The PICA frequently forms complex loops on the side of the brainstem, which distorts the medulla and the cranial nerves and results in various NVCSs such as glossopharyngeal neuralgia, hemi-laryngopharyngeal spasm, hemifacial spasm, trigeminal neuralgia, medullary compression syndrome, etc.
AVM	Posterior fossa AVMs associated diseases including aneurysm and dissection involving PICA should be focused because of their high risk of rupture, worse outcome and treatment options.
Surgical importance	The PICA is easily exposed and injured during surgeries especially associated with telovelar approach because of its special position, tortuous course and anatomical diversity. The PICA also plays an important role in bypass surgeries.
Other diseases	The PICA can be injured by head trauma, which causes fatal SAH and should be early diagnosed and treated. VA terminating in PICA probably cause BHS. The PICA supplies many brain tumors and can be used in intracerebellar chemotherapy.

PICA: posterior inferior cerebellar artery; VA: vertebral artery; CN: cranial nerve; EVT: endovascular treatment; LMS: Lateral medullar syndrome; rt-PA: recombinant tissue-type plasminogen activator; NVCS: Neurovascular compression Syndrome; AVM: Arteriovenous malformation; BHS: Bow hunter's syndrome;

## Abbreviations

PICA: posterior inferior cerebellar artery
NVCS: neurovascular compression syndrome
AVM: arteriovenous malformation
LMS: lateral medullary syndrome
BHS: Bow hunter's syndrome
VA: vertebral artery
CN Ⅸ: glossopharyngeal nerve
CN Ⅹ: vagus nerve
CN Ⅺ: accessory nerve
CN Ⅻ: hypoglossal nerve
CN Ⅶ: facial nerve
CN V: trigeminal nerve
BA: basilar artery
SCA: superior cerebellar artery
AICA: anterior inferior cerebellar artery
PPHA: primitive hypoglossal artery
PTA: primitive trigeminal artery
SAH: subarachnoid hemorrhage
IVH: intraventricular hemorrhage
MRA: magnetic resonance angiography
DSA: digital subtraction angiography
CTA: computed tomography angiography
EVT: endovascular treatment
mPICA: medial branch of the posterior inferior cerebellar artery
lPICA: lateral branch of the posterior inferior cerebellar artery
CT: computer tomography
MRI: magnetic resonance imaging
T2WI: T2-weighted images
FLAIR: fluid-attenuated inversion recovery
DWI: diffusion-weighted magnetic resonance imaging
HINTS: horizontal head impulse test, nystagmus and test of skew
rt-PA: recombinant tissue-type plasminogen activator
GPN: glossopharyngeal neuralgia
HeLPS: hemi-laryngopharyngeal spasm
HFS: hemifacial spasm
TN: trigeminal neuralgia
MCS: medullary compression syndrome
MVD: Microvascular decompression
IC-IC: intracranial-intracranial
EC-IC: extracranial-intracranial
OA: occipital artery
